# Malignant peripheral nerve sheath tumors (MPNST) – Clinicopathological study and treatment outcome of twenty-four cases

**DOI:** 10.1186/1477-7819-4-55

**Published:** 2006-08-22

**Authors:** Madhabananda Kar, SV Suryanarayana Deo, Nootan Kumar Shukla, Ajay Malik, Sidharth DattaGupta, Bidhu Kumar Mohanti, Sanjay Thulkar

**Affiliations:** 1 Oncology Unit, Department of Surgery, Himalayan Institute of Medical Sciences, Dehradun, India; 2 Departments of Surgical Oncology, Institute Rotary Cancer Hospital, All India Institute of Medical Sciences, New Delhi, India; 3 Radiation Oncology, Institute Rotary Cancer Hospital, All India Institute of Medical Sciences, New Delhi, India; 4 Pathology, Institute Rotary Cancer Hospital, All India Institute of Medical Sciences, New Delhi, India; 5 Radiodiagnosis, Institute Rotary Cancer Hospital, All India Institute of Medical Sciences, New Delhi, India

## Abstract

**Background:**

Malignant peripheral nerve sheath tumor (MPNST) is biologically an aggressive tumor for which the treatment of choice is the surgery. We reviewed the clinical profile, diagnostic methods, treatment patterns, and outcome of twenty-four MPNST patients in this study.

**Patients and methods:**

A retrospective analysis of 24 MPNST patients, treated from 1994 to 2002, in the department of Surgical Oncology at All India Institute of Medical Sciences, New Delhi, was done. A combination of gross, histopathological and immunohistochemical findings, and proliferation markers (MIB1) were considered for diagnosis and grade of the MPNST. Survival analysis was done by the Kaplan-Meier method and differences were evaluated with the log-rank test. Multivariate analysis was carried out by using Cox's proportional hazards model by using SPSS (Version 9, Chicago, Illinois) software.

**Results:**

MPNST constituted 12% of all soft tissue sarcomas, where 21% (5/24) of patients had associated Von Recklinghausen's disease (VRHD). A higher incidence of male preponderance and multifocal MPNST were noted in the present series. At a mean follow-up of 38 months, 13 (54 %) patients had relapse of disease and 5-year over all and disease free survival were 58% and 35% respectively. In univariate analysis, sex (*p *= 0.05), tumor depth (*p *< 0.03), and cellular differentiation (*p *< 0.002) were shown to be adverse prognostic factors for disease free survival and sex (*p *= 0.04), cellular differentiation (*p *< 0.0004), and tumor grade (*p *= 0.05) for overall survival. However, in multivariate analysis, cellular differentiation (*p *< 0.005) and tumor grade (*p *< 0.01) emerged as independent prognostic factors for both disease free and overall survival, respectively. Postoperative radiotherapy (RT) has shown a definite role in both disease free and overall survival in this study.

**Conclusion:**

MPNSTs constituted a significant proportion (12%) of soft tissue sarcoma in our medical center. Heterogeneous differentiation and multifocality of the tumor were few distinct features of MPNST. Sex and cellular differentiation were noticed as the new adverse prognostic factors and adjuvant radiotherapy has been proved to be a significant treatment tool in the current series.

## Background

Malignant peripheral nerve sheath tumor (MPNST) is a rare variety of soft tissue sarcoma of ectomesenchymal origin [1,2]. World Health organization (WHO) coined the term MPNST replacing previous heterogeneous and often confusing terminology, such as malignant schwannoma, malignant neurilemmoma, and neurofibrosarcoma, for tumors of neurogenic origin and similar biological behavior [3,4]. These tumors often create diagnostic problems because of their cellular origin and histopathological similarities with other spindle cell sarcomas like monophasic synovial sarcoma, leiomyosarcoma and fibrosarcoma [5]. They arise from a major or minor peripheral nerve branches [6] or sheath of peripheral nerve fibers [7,8]. These tumors may arise spontaneously in adult patients, although 5% to 42% of MPNST have an association with multiple neurofibromatosis Type-I [9-12]. Thus, a combination of gross, histopathological, and immunohistochemical studies are used for diagnosing these tumors. Another interesting clinical feature of this tumor is multifocality and development of second primary tumors of same histology [5]. Surgery is the main stay of treatment of this tumor though they are biologically aggressive in nature [5,12,13]. In this article, we reviewed the case records of the patients with malignant peripheral nerve sheath tumors to investigate their clinico-pathological features, treatment outcome, survival, and prognostic factors.

## Patients and methods

A retrospective analysis of MPNST patients treated in January 1994 to December 2002 in the Surgical Oncology unit at All India Institute of Medical Sciences, New Delhi was performed. The source of the data was a soft tissue sarcoma database. Twenty-four out of 200 soft tissue sarcoma patients had MPNST. The clinical details, including the presence or absence of VRHD [14], the histopathology, treatment details, and follow-up were analyzed. Pretreatment evaluation included core needle biopsy or incision and/or excision biopsy for the tissue diagnosis. Extent and stage of the disease was evaluated by the contrast enhanced computed tomography (CT) or magnetic resonance imaging (MRI). Patients with small (<5 cm) and subcutaneously located tumors underwent no imaging. All patients underwent surgical excision as the primary therapy and histopathological examination and immunohistochemical staining confirmed the final diagnosis. Adjuvant radiotherapy and chemotherapy was decided on the basis of the size (>5 cm), depth (deep seated), grade (high), and recurrence of the tumor. After completion of therapy all patients were followed-up every 3 monthly on regular basis. Survival was calculated from the date of diagnosis to the date of last follow-up or death. The details of local and systemic recurrences and second primaries were also analyzed. Development of a new sarcoma of same histology in a different anatomical area was taken as second primary or multifocal MPNST.

Survival analysis was done by the Kaplan-Meier method and differences were evaluated with the log-rank test. Multivariate analysis was carried out by using Cox's proportional hazards model.

## Results

MPNST constituted 12% (24/200) of all soft tissue sarcomas. Out of 24 patients, 19 were male and 5 were female (M:F = 3.8:1). The age ranged from 16 to 81 years, with median age of 40 years. Five out of 24 (21 %) patients had an associated Von Reckling Hausen's disease. Three patients had plexiform variety among five patients with multiple neurofibromatosis. The commonest presenting symptom was a "mass" (96%) followed by pain (71%). Only one patient had neurological deficit. Extremities were the commonest site involved (15/24), followed by chest wall, trunk, pelvis, and head and neck (Table 1). CT scan was performed in 6, MRI in 9, and both CT and MRI scan were performed in 3 patients. Out of 24 patients, 22 (92%) were deep seated and two (8%) were superficial tumors. Only in eight cases the neural origin of tumors could be identified intra-operatively (4 in lower limb and 3 in upper limb and one in pelvis), while in the rest, it was not possible to specifically identify the nerve of origin.

**Table 1:** Anatomical site distribution of the MPNST.

**Site of Tumor**	**Patients (Percentage)**
Head and neck	01 (04%)
Chest wall & trunk	06 (25%)
Extremity	15 (63%)
Upper limb	07 (29.2%)
Lower limb	08 (33.8%)
Pelvis	02 (08%)

### Pathology

The following criteria were used for the histological diagnosis of MPNST – a) gross fusiform tumors in relation to nerves, b) microscopic features of spindle cell with fascicular pattern and varying degrees of mitosis, necrosis and tumor calcification, c) presence of associated benign neurofibroma or schwannanian cells, and d) positive immunohistochemical staining for S-100 protein, neuron specific enolase (NSE) and others like actin, cytokeratin (CK), smooth muscle actins (SMA), desmin, and vimentin to differentiate from other spindle cell sarcomas. The tumors were classified as low and high grade on the basis of their cellular differentiation, mitotic count, tumor necrosis [15,16] and expression of MIB-1 proliferation marker [17,18]. Tumor necrosis was evaluated with scoring as 0, 1, and 2, depending on the percentage of necrosis as 0%, <50%, and >50% presence, respectively. Mitosis rate is also evaluated like wise as 0, 1, and 2, depending on numbers as <5, 5–10, and >10 per 10 hyper power field (HPF), respectively. More than 5 mitotic rates per 10 HPF have been considered as high grade tumor as single mitotic figure may be significant in a tumor with hypercellularity and nuclear atypia [13]. The significance of the mitoses depends on the prognostic value of increased cell proliferation. Tumor necrosis scoring was 0 in 14 patients, 1 in 6 and 2 in 4 patients, respectively, while mitotic rate was 0 in 4, 1 in 8, and 2 in 12 patients, respectively. A >5% cellular staining of MIB-1 proliferation marker has been considered as high grade tumors [17,18]. The MIB-1 proliferation marker has been done in twenty cases, where >5% staining of cells shown a correlation with the high-grade tumors, while <5% had shown low grade MPNST. Preoperative tissue diagnosis of malignant nerve sheath tumor could be made in 15 (62.5%) patients on biopsy specimens and the remaining were diagnosed as unclassified malignant mesenchymal tumors. In the final histopathology, all patients were diagnosed as malignant peripheral nerve sheath tumor. S-100 immunohistochemical stains were used in twenty cases, of these, fifteen cases (63%) showed strong focal positivity. Other cases were supported by exclusion method of other soft tissue sarcomas by various other immunohistochemical stains. Fifteen patients (62.5%) were categorized as having high-grade and the remaining 9 (37.5%) as low-grade MPNST, on the basis of above pathological parameters. A peculiar proliferation of tumor in the sub-endothelial zones of vessels with neoplastic cells herniation into vessel lumen and proliferation of small vessels in the walls of large vessels have been noticed in our series, which are very characteristic features to designate the tumor as MPNST. In our study, five patients (21%) have shown divergent differentiation of MPNST with heterotrophic such as rhabdoid, chondroid, myoid, epitheloid and osteoid elements in each case, respectively. We encountered some rare pathological findings, such as skeletal muscle entrapment and squamous differentiation, in our series. Size of the tumor ranged from 4 to 24 cm in greatest diameter (mean 10.83 cm), where twenty-two had more than 5 cm size and 8 had more than 10 cm tumor. Two patients had positive tumor resection margin after surgery.

### Treatment

Curative surgery was performed in all 24 cases in the form of wide local excision in 17 cases, amputation or disarticulation in 5 cases and two patients had pelvic exenteration for large pelvic MPNST. Among the 15 patients with extremity MPNST limb salvage surgery was performed in 10, and the remaining 5 had amputations or disarticulation either for primary or recurrent tumor with neuro-vascular encasement and extensive soft tissue with bone involvement. In the current study, 8 out of 24 patients required radical surgery and 16 required conservative surgical procedures. The overall limb salvage rate was 67%. In view of the size (>5 cm), location, and grade, postoperative adjuvant radiotherapy, ranging from 54 to 62 Gy (median dose 58 Gy), was given to 16 patients. Three patients in the radical surgery group and 13 in the conservative surgery group received postoperative radiotherapy. One patient with a Head and Neck MPNST, which was of rhabdoid differentiation (malignant triton tumor), was treated by commando operation and given adjuvant postoperative radiotherapy and chemotherapy. Two patients had disarticulation for their regional recurrence of the disease, with one of these finally succumbing to systemic relapse. All systemic relapse patients received palliative chemotherapy, as no patient was suitable for curative resection.

### Survival

**Figure 1:**
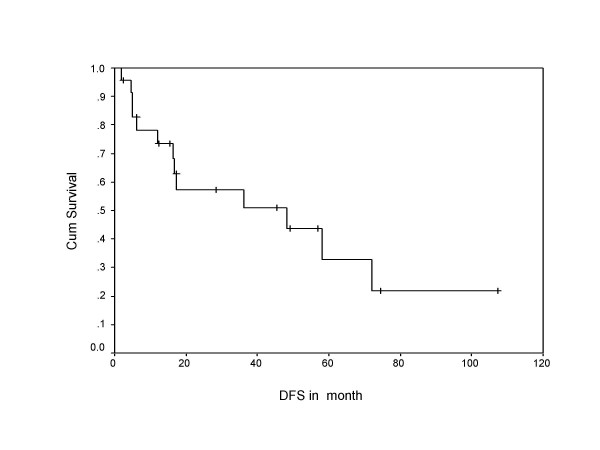
Shows Kaplan Meier survival curve indicating disease free survival in months.

**Figure 2:**
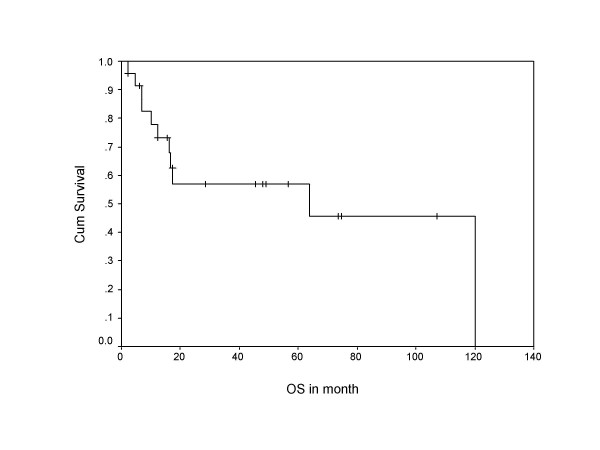
Shows Kaplan Meier survival curve indicating overall survival in months.

**Figure 3:**
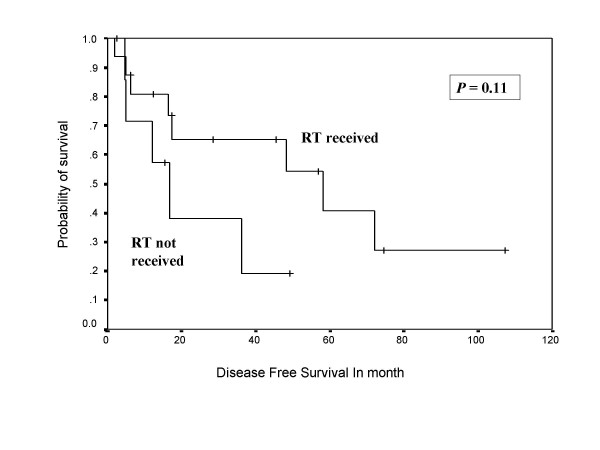
Shows Kaplan Meier Curve indicating the effect of postoperative radiotherapy on disease free survival.

**Figure 4:**
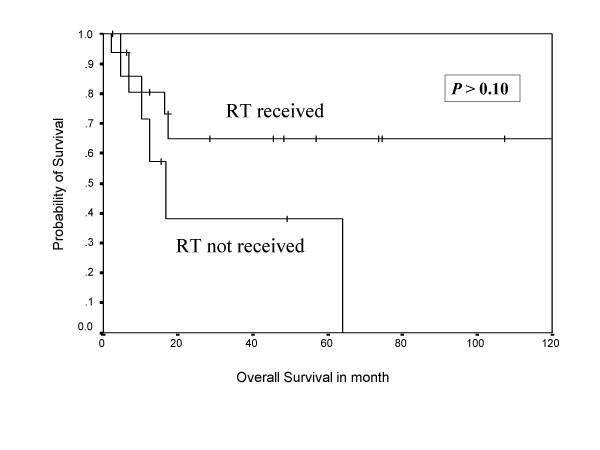
Shows Kaplan Meier Curve indicating the effect of post operative radiotherapy on overall survival.

During follow-up, 13 (54 %) patients developed relapse of the disease, including local, systemic, and second primary sarcomas of same histology (multifocal MPNST). Both tumor positive resected margin patients developed systemic relapse of the disease, including one with local recurrence. Overall 10 patients (around 77% of all recurrences) developed systemic relapse, with lung (7 out of 10 = 70%) being the commonest site, while liver, spleen, spine, and orbit were other systemic sites noted for rest of the cases. Eighty percent of systemic relapses (8 out of 10) occurred within two years of their treatment. The 5-year overall and disease free survivals were 58% and 38%, respectively, with median disease free period was 17 months (Figure 1, 2), where as median survival period was 32 months. Ten out of 24 patients (41%) died due to disease, one patient with multiple neurofibromatosis died at the age of 87 not due to disease and the remaining were alive and disease free. Patients who had not received post operative RT had no 5 year disease free survival, while 42% patients who received RT were disease free after 5 years in the current series in Kaplan Meier survival curve. Similarly, 65% patients received postoperative RT had 5 year overall survival versus only 38% patients not received RT. (Figure 3, 4).

### Prognostic factors

Sex, tumor depth, VRHD, tumor necrosis, mitotic rate, cellular differentiation, and tumor grade, heterogeneous differentiation of tumor, radiotherapy treatment, and relapse of the disease were studied for prognostic factors.

**Figure 5:**
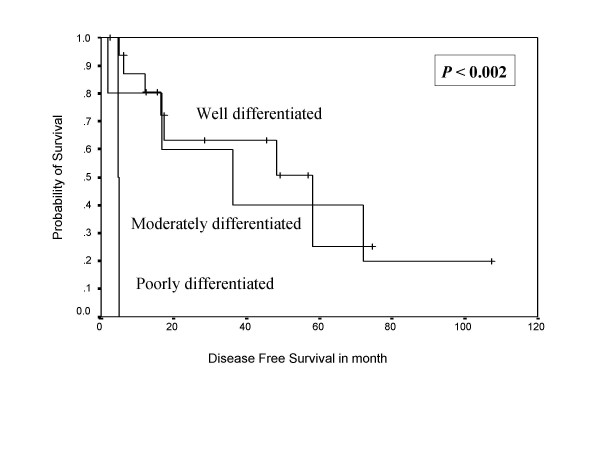
Kaplan Meier Curve indicating the impact of cellular differentiation on disease free survival.

**Figure 6:**
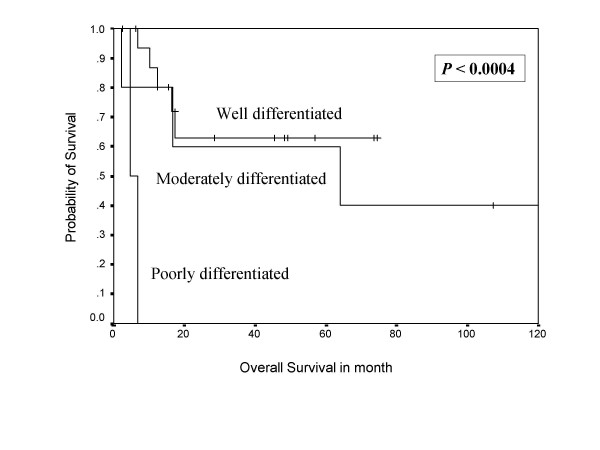
Kaplan Meier Curve indicating the impact of cellular differentiation on overall survival.

In univariate analysis, sex, tumor depth, and cellular differentiation were shown to be adverse prognostic factors for the disease free survival, while sex, cellular differentiation (Figure 5, 6), tumor grade, and relapse of the disease were shown to be negative prognostic factors for overall survival (Table 2). In multivariate analysis, cellular differentiation and tumor grade emerged as independent adverse prognostic factors for  disease free and overall survival respectively (Table 3). VRHD association, tumor necrosis, and mitosis rate did not have any impact on survival.

**Table 2:** Prognostic factors affecting disease free survival and overall survival in univariate analysis.

**Variables**	**Chi Square**	**95% C.I.**	***P *Value**
**DFS**			
1. Sex	3.84	0.92 – 12.93	0.05*
2. Tumor depth	4.23	5.3 – 9.4	<0.03*
3. VRHD	0.28	0.28 – 3.9	0.59
4. Tumour Necrosis	0.56	0.2 – 10.4	<0.76
5. Tumor mitosis	0.027	0.22–3.53	>0.86
6. Cellular differentiation	11.95	2.01 – 111.75	<0.002*
7. Tumor grade	1.69	0.14 – 1.51	>0.19
8. Heterogeneous differentiation	1.73	0.14 – 1.48	0.18
9. RT	2.49	0.76 – 8.42	0.11
**OS**			
1. Sex	4.02	0.94 – 13.27	0.04*
2. Tumor depth	1.86	0.4 – 1.18	0.17
3. VRHD	0.15	0.18 – 4.08	>0.37
4. Tumor Necrosis	0.6	0.2–14.2	>0.74
5. Tumor Mitosis	0.012	0.2–4.8	>0.98
6. Cellular differentiation	15.50	2.44 – 334.28	<0.0004*
7. Tumor grade	3.84	0.94 – 4.68	0.05*
8. Heterogeneous differentiation	0.89	0.15 – 1.95	0.34
9. RT	2.66	0.10 – 1.28	>0.10
10. Recurrence	9.49	0.3 – 2.61	<0.002*

C.I. = Confidence Interval, DFS = Disease free survival, OS = Overall survival, VRHD = Von Reckling hausen's disease, RT = Radiotherapy, *: Statistically Significant.

**Table 3:** Prognostic factors affecting disease free survival and overall survival in multivariate analysis.

**Variables**	**Chi Square**	**95% C.I.**	**Hazard Ratio**	***P *Value**
**DFS**				
1. Cellular differentiation	10.51	4.20 – 463.30	0.3101	0.005*
**OS**				
1. Tumor Grade	6.08	1.41 – 3.07	0.2741	0.01*

C.I. = Confidence Interval, DFS = Disease free survival, OS = Overall survival, *: Statistically Significant.

## Discussion

MPNST is a very rare tumor, with an incidence of 1 per 1,00,000 population and whichconstitutes between 3 to 10% of all soft tissue sarcomas. Hence, this entity is often managed as a sub-category of soft tissue sarcomas [1,2]. In contrast, in our series, MPNST constituted 12% (24/200) of all soft tissue sarcomas, with MPNST representing the second most common variety of soft tissue sarcoma seen. The most significant contributions in understanding the clinical and pathological features of MPNST were studied by Mayo clinic investigators [6,16,19]. A combination of gross and microscopic findings along with immunohistochemical studies is commonly used to diagnose a case of MPNST [5]. In most instances, the tumors display fascicles of spindle cells woven into herringbone pattern with varying degrees of mitosis and necrosis. However, it is not always possible to demonstrate the origin from a nerve, especially when it arises from a small peripheral branch. This point was exemplified in a series by Nambisan *et al*., in which nerves could not be identified in 61% of cases of MPNST [12] and in the series Bilge *et al*., in which nerve origin could be identified only in 45–56% cases [20]. Still, there are several other distinct features, such as proliferation of tumor in the sub-endothelial zones of vessels with nepotistic cells herniation into vessel lumen and proliferation of small vessels in the walls of the large vessels, which are very characteristic features of MPNST [21], as noticed in our series.

In our series, grade of the tumor emerged as the significant prognostic factor for overall survival. Likewise, cellular differentiation emerged as a significant prognostic factor for disease free survival in both univariate and multivariate analysis. However, other pathological findings, such as mitosis and tumor necrosis, had no impact on survival in our series. In our series, the index of increasing proliferation, MIB-1, was clearly correlated with grade of the tumor and simultaneously with prognosis of the disease. Ducatman *et al*. [5] reported that tumor grade and mitotic count were of no prognostic utility, while Enzinger *et al*. [21] noticed that grade of the tumor, necrosis, vascular invasion, and presence of mitosis have significant influence on survival of the patient. We have 21% of patients who have heterotrophic pathological features of the tumor; however, such divergent differentiation did not affect the survival (Table 2). In contrast, Ramanathan *et al*. [22] and Scheithauer *et al*. [23] have reported the divergent differentiation as a significant adverse prognostic marker for MPNST in their series.

These tumors occur with equal frequency in males and females and some series have shown a female preponderance [6,7]. However, we found a significant male preponderance (80 %) in the current study and this significantly influenced the disease free (*p *= 0.05), as well as overall survival (*p *= 0.04). But it is very difficult to draw any conclusion about sex as significant prognostic factor due to small series and it may be due to referral bias. As far as the site distribution was concerned. The majority had involvement of the extremities, although tumors were also seen in unusual sites, such as the pelvic retroperitoneum and infratemporal fossa. In our series, the site of the tumor had no impact on survival of the patients (extremity versus others was p < 0.06).

The association of MPNSTs with VRHD is well known [7,8,23] and the series reported 5% to 42% neurofibromatosis patients develop sarcomas. In a review of 71 years experience, Ducatman et al., [5] estimated that the risk of developing MPNST in VRHD to be 4600 times greater than the general population. In the current study, 21 % of patients had clinical features of VRHD, but their association did not affect the survival of the patients (Table 2), which is in contrast to the findings of other authors [1,5,24]. In fact, the longest surviving patient in our series was a patient having multiple neurofibromatosis who presented with 14 malignant transformations of benign neurofibromas over an 8-year period.

Routine preoperative electrophysiological examination is not integral to the management of MPNST. This is so since such an examination neither contributes to the diagnosis nor influences the treatment plan [2]. Imaging is routinely performed to assess the extent of the disease and plan surgical resection. However, it does not reliably determine the malignant transformation from neurofibroma to MPNST [13]. A target lesion in T2MR image is an indication of low grade while heterogenous lesion due to necrosis & hemorrhage and patchy contrast enhancement in MRI is an indication of malignant MPNST [25,26]. MRI is the investigation of choice because it can reveal the nerve of origin and its relationship to adjacent structures [27]. Clinical behavior of the disease with radiological correlation can also guide to plan the treatment. More importantly, we used contrast enhanced computed tomography (CECT) for assessment of pulmonary metastasis, where MRI has limitations, and also for some primary cases, when MRI was not available initially at our institute.

**Figure 7:**
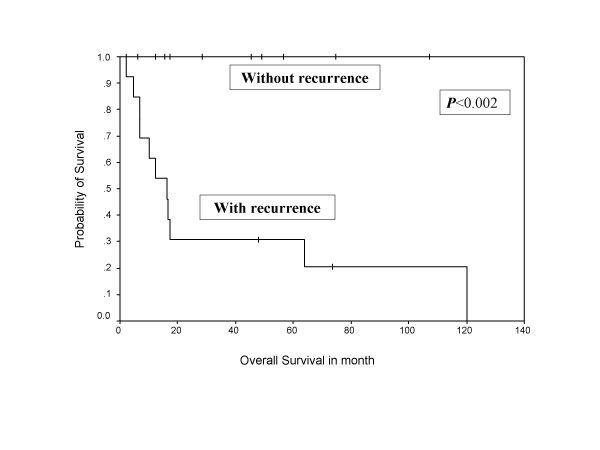
Shows Kaplan Meier projected survival curve indicating the influence of relapse of the disease on overall survival of the patients.

Radical surgical resection is the treatment of choice in MPNST. A good three-dimensional clearance is mandatory for a successful outcome. Amputations are indicated only when wide excision is not feasible and in patients with severely compromised limb function. Routine nodal dissection is not indicated. However, when a major nerve is identified, the cut end should be sent for frozen section to assess the tumor free margin of the resection. MPNSTs are generally considered chemotherapy and radiotherapy resistant tumors. However, there are reports of routine postoperative radiotherapy and even radiotherapy as a single modality alone for MPNST in literature [28]. In view of the rarity of this entity and conflicting reports, it is difficult to define the role of radiation in the management of MPNSTs. Currently, postoperative radiotherapy is recommended by oncology consensus group [13] as part of a uniform treatment policy for MPNSTs, much like other high grade soft tissue sarcomas [3,9], despite having clear surgical margins. Basso-Ricci [29] demonstrated 56% disease free survival using combined surgery and radiation therapy for MPNST. Although adjuvant radiotherapy has not been cited as a significant prognostic factor (Table-2) in the current study, the results demonstrated in Figure 7 and 8 do reveal a trend towards an impact on both disease free and overall survival. The indication of radiation treatment are biased towards patients having tumors with poor prognosis (high-grade or recurrent, deep seated, and bigger size), and thus failing to show the statistically significant difference between radiated and non-radiated patients.

MPNST has the highest recurrence rate of any sarcomas [30], and adequate initial treatment gives the best chance of survival [28]. In the current series, recurrence after initial treatment, whether local or metastatic, has been cited as a poor prognostic factor for overall survival (P < 0.002, Figure 7). The most important feature in present study was the nature of the disease relapse due to which even one patient could not be surgically salvaged and were treated with palliative chemotherapy.

## Conclusion

MPNST constitutes a significant proportion of soft tissue sarcoma in our study. A combination of clinical, pathological, and immunohistochemistry helps in diagnosing these tumors. Proliferation marker (MIB1) can be a good adjunct to grade and tailor the treatment in MPNST. The overall treatment approach should be like that of any other high grade sarcomas. Heterogeneous differentiation and multifocality of the tumor were few distinct features of MPNST in our series. Sex and cellular differentiation emerged as the new adverse prognostic factors for survival of the patients, where as VHRD association had no impact on survival in our study. Postoperative radiotherapy has a definite role in both disease free and overall survival. Though multimodality therapy, including surgical resection and adjuvant radiotherapy, is available, the prognosis remains dismal. Modern clinical studies and the development of effective targeted chemotherapy are needed to gain control of the disease.

## Competing interests

The author(s) declare that they have no competing interests.

## Authors' contributions

**MK **– Conceived the study, design, acquisition of data, analysis, and interpretation of data.

**SVD **– Conceived the study and participated in its design and coordination and revising it critically for important intellectual content.

**NK **– Revising it critically for important intellectual content.

**AK **– Carried out the histopathology, immunoassays and proliferation marker staining.

**SDG **– Carried out, analysis and interpretation of the histopathology and immunoassays.

**BM **– Revising it critically for important intellectual content.

**ST **– Carried out the imaging and its critical interpretation.

All authors read and approved the final manuscript.
